# A descriptive phenomenology study of newcomers’ experience of maternity care services: Chinese women’s perspectives

**DOI:** 10.1186/1472-6963-14-114

**Published:** 2014-03-07

**Authors:** Tsorng-Yeh Lee, Christine Kurtz Landy, Olive Wahoush, Nazilla Khanlou, Yin-Chun Liu, Chia-Chi Li

**Affiliations:** 1 School of Nursing, York University, #312 HNES Building, 4700 Keele St, Toronto, ON M3J 1P3, Canada; 2 School of Nursing, McMaster University, 1280 Main Street West, Hamilton, Ontario L8S4L8, Canada; 3 Women's Mental Health Research, Faculty of Health, Academic Lead, Lillian Wright Maternal Child Health Scholars Program, School of Nursing, York University, 270G York Lanes, 4700 Keele St., Toronto, ON M3J 1P3, Canada; 4 National Yang-Ming University, No.161, Sec. 6, Minquan E. Rd., Neihu Dist., Taipei City 114, Taiwan; 5 School of Nursing, National Defense Medical Center, No.161, Sec. 6, Minquan E. Rd., Neihu Dist., Taipei City 114, Taiwan

**Keywords:** Access to maternity health services, Chinese, Women, Immigrants

## Abstract

**Background:**

Maternity health care available in Canada is based on the needs of women born in Canada and often lacks the flexibility to meet the needs of immigrant women. The purpose of this study was to explore immigrant Chinese women’s experiences in accessing maternity care, the utilization of maternity health services, and the obstacles they perceived in Canada.

**Methods:**

This descriptive phenomenology study used in-depth semi-structured interviews to examine immigrant Chinese women’s experiences. Fifteen participants were recruited from the Chinese community in Toronto, Canada by using purposive sampling. The interviews were digitally recorded and transcribed verbatim into written Chinese. The transcripts were analyzed using Colaizzi’s (1978) phenomenological method.

**Results:**

Six themes were extracted from the interviews: (1) preference for linguistically and culturally competent healthcare providers, with obstetricians over midwives, (2) strategies to deal with the inconvenience of the Canadian healthcare system (3) multiple resources to obtain pregnancy information, (4) the merits of the Canadian healthcare system, (5) the need for culturally sensitive care, and (6) the emergence of alternative supports and the use of private services.

**Conclusions:**

The findings provide new knowledge and understanding of immigrant Chinese women’s experiences in accessing maternity health services within a large metropolitan Canadian city. Participants described two unique experiences within the themes: preference for linguistically and culturally competent healthcare providers, with obstetricians over midwives, and the emergence of alternative supports and the use of private services. Few studies of immigrant maternity service access have identified these experiences which may be linked to cultural difference. Further investigation with women from different cultural backgrounds is needed to develop a comprehensive understanding of immigrant women’s experiences with maternity care.

## Background

Maternity care available in Canada is largely based on the needs of women born in Canada and often lacks the flexibility required to meet the needs of immigrant women [1-4]. Immigrant women experience many challenges that can make access to maternity care difficult. The barriers to health services access include language and cultural barriers, social isolation, family separation, low income, poor housing and work schedules [1,5-9]. Inadequate maternity care can have deleterious effects on women’s health such as maternal anemia and postpartum depression, and on the fetuses and newborns’ health with higher infant mortality or prematurity [10-12]. Access to high quality maternal care is important for the health of women and their newborns.

In Canada, Chinese immigrants are the second largest immigrant population. According to the 2011 National Household survey, there are over 1,324,700 Chinese immigrants living in Canada. They make up 21.1% of the visible minorities living in Canada [13]. Chinese immigration has a very long and possibly the longest history of migration to Canada. However, little attention has been paid to migrant health in general [2].

Peters et al. [14] in 2008 described four dimensions of access to health services: geographic accessibility, availability, financial accessibility and acceptability. These four dimensions have important impacts on immigrants’ use of health services. One of the biggest challenges in health services delivery in Canada is the availability and acceptability of maternity care that is sensitive and accommodating to the needs of immigrant women. Language, culture and ethnicity are important factors that influence immigrants’ choice of health care provider and health management strategies in the host country [15,16]. An American study of 52 Chinese immigrants found that cultural factors, i.e. beliefs about health, health care and illness were strongly associated with access to and utilization of health services [17]. The study also reported that communication and mistrust in Western health care affected utilization of health care among mainland Chinese and Taiwanese. During pregnancy and childbirth these language, cultural and ethnic factors create additional barriers to health service access and utilization. A Canadian study of pregnant immigrant Muslim women revealed that they received little culturally and linguistically appropriate information about pregnancy and childbirth [1]. They also reported that maternity care providers did not consider their religious needs which created barriers to maternity health services access [1]. For some women these barriers caused them to delay use of maternity health services until delivery [1]. The need for maternity care providers and services to accommodate cultural, religious and ethnic diversity may not only be important to pregnant immigrants who are new to their country of residences but also to immigrants who appear acculturated in the host country. Lagana found that immigrant women returned to many of their traditional practices once pregnant no matter how long they had lived in their host country [18].

Immigrants to Canada often prefer to use health services providers who speak their language. Wang et al. found that in Toronto immigrants from mainland China preferred to access family physicians who were linguistically and ethnically matched to their own background and were willing to travel to access these physicians instead of physicians closer to home [15]. No published studies were found that specifically examined whether pregnant immigrant Chinese women in Toronto had the same preference. Interestingly in Toronto 5.4% of obstetricians can provide care in the Chinese language [19].

Many women from China come with the belief that obstetricians provide superior maternity care compared to other providers and do not want midwifery care [20]. Cheung et al. who investigated mothers’ attitudes toward midwives in China reported that the mothers were unsatisfied with midwives’ skills [20]. They also found that the profession of midwifery was viewed poorly by the general public. In recent years the emergence of medical insurance, the favorable transformation of public attitudes towards hospital birth and the widespread use of Caesarean sections have led Chinese women to prefer obstetricians [21-23]. In contrast a recent pan Canadian study found that women in Canada are most satisfied with the care received from midwives, compared to obstetricians and family physicians [24].

Social support is very important to childbearing immigrant women and is intricately connected with health [25-27]. Appropriate levels of social support decrease the impact of stressful life events, facilitate healthy childbirth transition and are linked to postpartum women’s health status [25,28-31]. Childbearing women who lack adequate support are at higher risk for mental health challenges and report poorer overall health [6,25,27]. Childbearing immigrant women, particularly during the early years in Canada are at higher risk for poor health outcomes than women born in Canada likely because they often experience low levels of social support (including access to financial support) and have smaller social networks than women born in Canada [6,7,25,27]. For Chinese women social support is critical if they are to implement their cultural practice of “doing the month”. In Chinese society “doing the month” is a historical tradition which provides mothers with emotional, informational and instrumental support [32]. There is a paucity of research examining immigrant childbearing Chinese women’s experiences of social support that protects against stressors and challenges that occur during the postpartum period [33]. Traditionally the mother’s mother or mother in-law provides the support required to “doing the month” [34]. There is some evidence that some immigrant women who wish to “doing the month” encounter governmental barriers to bringing family members to Canada to provide needed support. In one Canadian study postpartum immigrant women reported that their family members were often unable to obtain timely visitor visas and thus had no one to help them after their infants were born [7].

While many studies were focused on the barriers of maternity health access for immigrant pregnant women or comparison the differences in pregnant outcome of Canada-born and foreign-born women, these studies have mostly used quantitative methodological approaches. Few qualitative studies have exclusively explored the experiences of immigrant women and their concerns regarding access to maternity health care in Canada [1]. This manuscript reports the findings of a descriptive phenomenology study examining childbearing immigrant Chinese women’s health service needs and utilization patterns in Canada. The purpose of this study was to explore immigrant Chinese women’s experiences in accessing and utilizing maternity health services, and the barriers they perceived in getting care in Toronto, a large Canadian metropolitan city in which 40% of the population is born outside of Canada [13]. The research questions were: a) what are Chinese immigrant women’s experiences when they access maternity health care services during pregnancy, labour and delivery, and postpartum? And b) what kinds of factors facilitate and hinder their access to maternity care?

## Methods

This descriptive phenomenology study used in-depth semi-structured one-on-one interviews to examine immigrant Chinese women’s experiences accessing and using maternity care in Toronto, Canada [35]. The study was approved by York University’s research ethics board. Chinese women were eligible to participate if they: 1. immigrated to Canada within the last 10 years; 2. resided within the Greater Toronto area; 3. spoke fluent Mandarin; and 4. gave birth within the last two to six months. Using purposive sampling participants were recruited from community health care centres and prenatal and postnatal classes. Recruitment flyers promoting the study were placed on the bulletin boards at these locations. Potential participants were contacted by TY by phone after they left their contact information to the service providers at the above mentioned locations. A home visit was arranged if they agreed to participate in the study. Consent was signed after the study purpose and procedures were explained to each participant. Recruitment of participants continued until no new information emerged from the interview data. Participants received $ 25 each in appreciation for their participation in this study.

Data were collected between May 2011 and July 2012. Participants were interviewed in Mandarin at their homes by TY who is fluent in Mandarin. The open-ended interview guide was developed by the authors of this study based on their knowledge on this area and included questions such as: “Please tell me how you accessed maternity health care when you were pregnant?” “What kinds of things helped you access/hindered you access to maternity care?” and “How do you feel you were treated by the maternity care providers/system?” and “How do you feel about the quality of the care you received?” Interviews lasted 90 to 120 minutes, were digitally audio recorded and transcribed verbatim in Mandarin. Transcripts were stripped off identifying data and replaced with a participant ID number to ensure confidentiality.

In a descriptive phenomenology, the researchers often bring their perspectives, experiences, values, beliefs, and identity to the data collection and analysis process. In order to reduce the subjective elements, data were analyzed by a group of three researchers: TY, YC and CC who spoke the same language as the participants (Mandarin). Colaizzi’s seven-step phenomenological approach was used to analyze the interviewed data [36]. The seven steps were as follows: 1. Each participant’s transcript was read by the three authors to achieve a deep understanding of the description and make sense of it. 2. Each individual transcript was reread by the three authors and phrases that directly relate to the phenomenon under investigation were extracted, such as the women’s specific experiences. 3. Formulated meanings of each significant statement were created. 4. The three researchers repeated these steps for each transcript and then aggregated formulated meanings into clusters of themes. 5. Six themes were identified and an exhaustive description was developed. 6. The essential structure of the description of the experience was identified. Finally, the essential structure was validated by the participants. The three authors met regularly in the summer of 2012 to discuss and verify the accuracy of the emerging themes and the meaning of each theme. A theme was not accepted until agreement by three authors was reached 100% of the time. Member checking was used to ensure the rigor of the analysis. The themes that emerged capturing the women’s experiences were sent back through emails to all the participants for verification and confirmation of the researchers’ interpretation of their experiences [35,36]. All of the participants agreed with the interpretation. TY translated the study results into English in order to allow for broad dissemination of the findings. The RATs checklist was used to report this qualitative study [37].

## Results

Fifteen participants were recruited and participated in the one-on-one in-depth interviews. Ten of the participants were originally from China; three were from Hong Kong and two were from Taiwan. The mean age of the participants was 33.4. The majority of the participants were married and unemployed. Most of them had a university degree. Two-thirds were religious believers, including Buddhists, Christians and Taoists. While 13 participants had their husband present during birth, the key helper during postpartum period varied, including husband, own mother, mother-in-law or “Yue-Sao” (the postpartum support provider).

The following six major themes emerged from the findings: (1) preference for linguistically and culturally competent healthcare providers, with obstetricians over midwives, (2) strategies to deal with the inconvenience of the Canadian healthcare system, (3) multiple resources to obtain pregnancy information, (4) the merits of the Canadian healthcare system, (5) the need for culturally sensitive care, and (6) the emergence of alternative supports and the use of private services.

### Preference for linguistically and culturally competent healthcare providers, with obstetricians over midwives

The women in this study wanted to be cared for by health care providers who spoke their language and were knowledgeable about their culture. Their first choice for maternity care provider was Chinese-speaking obstetricians followed by Chinese-speaking midwives. English-speaking healthcare providers were their last choice.

Eleven women chose obstetricians who spoke Chinese Mandarin or Cantonese because they felt that it would be easier for them to communicate with their obstetrician and that they shared the same cultural beliefs about maternity care. Only two of the 15 participants received care from English speaking obstetricians. They explained that all of the Chinese speaking obstetricians in their area were not accepting new patients. One of these participants shared that although her English was good enough to communicate with her obstetrician, she still felt a communication barrier existed between her and her obstetrician. She stated that she only asked simple questions and if the obstetrician were Chinese, she would have asked much deeper questions.

Two participants chose a Mandarin-speaking midwife because they started their antenatal care in the second trimester. They expressed that they preferred an obstetrician over a midwife at the beginning of their pregnancy because they were afraid a midwife would not be able to provide good care especially if there were complications. One of these women shared: *“We don’t choose a midwife back home. I am afraid if I need a caesarean section, a midwife can’t do the operation. If I had a choice, I wouldn’t use a midwife.”* The other participant stated: *“Midwives have never been one of my choices to deliver my baby.”* However, after being rejected by several obstetricians, they thought that a Mandarin-speaking midwife might be a good option because at least the communication with the midwife would be easier than that of an English-speaking doctor. This was highlighted by one participant’s comments: *“I am worried about the process of delivery. I feel safe if someone can explain to me what’s going on using the language I understand.”* Despite the two participants’ initial reluctance to use a midwife they were very positive about the care they received. They liked the fact that the midwives were punctual at appointments and had caring attitudes. In addition, they liked the continuity of the care provider, i.e. the same midwife care for them throughout the pregnancy, birth and postpartum.

### Strategies to deal with the inconvenience of the Canadian healthcare system

Participants shared that the Canadian health care system is quite different from the system in their country of birth. For example, they shared that they needed a family physician’s referral to see an obstetrician in Canada, whereas in their country of birth they had the freedom to self-refer, and to choose when and where to visit any doctor who best suited to meet their needs. Participants explained that in order to overcome this inconvenience in Canada they used the advice of friends and internet sites of “the top or best obstetrician” to identify “suitable” obstetricians to take care of them. Participant 6, for example, explained: *“I phoned the OB’s clinic which I preferred. After confirming they had openings for new patients, I then requested my family physician to refer me to that OB.”*

Participants had to endure several inconveniences in order to access to the obstetrician they preferred. Most women travelled less than 45 minutes to reach their obstetrician’s office; but some travelled more than an hour. Participant 15 shared: *“My doctor’s office is a little bit far away from my home. I need to transfer twice (two buses and one subway) and walk about 10 minutes to get there. Sometimes I felt tired.”* Another inconvenience participants noted was the state of their obstetricians’ waiting rooms. Many shared that the waiting rooms were so crowded with patients that they were unable to find a seat even though they felt very tired. This was made even worse by the long wait times to see their obstetricians. Participant 8 described: *“There is always a long waiting line. I have to wait in the OB’s clinic for two hours in order to see my OB. It is so crowded there. I sometimes have to stand outside the clinic.”*

Some participants discussed the problem of lack of continuity of maternity care provider through their pregnancy and at the birth. They wanted to have the obstetrician that cared for them throughout their pregnancy to attend their birth. Participant 4 stated: “*My OB wasn’t on duty and I had another OB that was ‘on call’ and he did not know anything about me and my pregnancy. It was difficult, especially, I had DM (diabetic mellitus) during pregnancy and my labour lasted sixteen hours.”* Participant 4 felt that the doctor who was on call did not help much. She needed to start to retell her history to the obstetrician on duty in order to receive proper care. Another participant who had her first child delivered in her country of birth mentioned that *“Back home, you can get the same OB to deliver your child as long as you pay the ‘appointed labour and delivery fee’ or give the OB a red envelop with money.”* Several participants mentioned that their obstetricians understood this custom and were willing to help them; however, it did not guarantee that the same obstetrician would be present at their birth. Participant 6, for example, stated: *“My OB told me if he is on call on that day when I am in labour; he will assist me to give birth. But if he is not on call he will only help me until midnight.”* Participant 6’s OB helped her to deliver her baby because it was at eight o’clock in the morning even though he was not on duty on that day. Many participants suggested an after hour’s payment to the obstetrician could ensure their own doctor would attend their births and thus ensure continuity of care providers.

### Multiple resources to obtain pregnancy information

Participants identified multiple resources where they could obtain pregnancy related information, such as the obstetrician’s clinic, community health centres, prenatal classes, parents, friends and the internet. Most of the participants reported receiving some information on physical and psychological pregnancy related changes, nutrition and exercise either verbally from their obstetrician or in the form of English or Chinese pamphlets in the obstetrician’s office. The pamphlets were placed on the bookcase in the waiting room of the obstetricians’ clinics which made it convenient for them to pick up the information they needed. However, participants shared that the information received at the obstetrician’s office or from the obstetrician was often not enough to address their concerns and answer their questions related to pregnancy. They reported accessing additional needed information from the community. For example, Participant 8 stated:

My OB didn’t provide me with much pregnancy related information. The nurse over there [in the doctor’s office] can only speak English. My English is not good and I didn’t know how to ask a question in English. But I can get similar information from the community health centre. There are several community health centres that provide such information in Chinese.

Many of the participants attended prenatal classes to get needed education. Half of the participants attended private Chinese-speaking prenatal classes which consisted of seven two-hour classes taught by hospital nurses. Other participants attended hospital based or public health department based English-speaking prenatal classes which consisted of four two-hour classes. Participants could repeatedly attend these four classes as needed. Participant 4 attended a diabetic class to help her learn how to control her high blood sugar. Most participants valued the information taught in the classes stating it was very practical and useful. Some participants who attended the English prenatal classes shared that they were provided with Chinese interpreters who translated the English content into Chinese which they found extremely helpful.

Participants also got needed information from their parents and/or friends through local and/or international phone calls. In addition many participants reported using the internet to get needed information. For example one participant stated she used the internet to get information on practical techniques to breastfeed her baby. The women accessed Chinese websites and preferred Chinese websites to get most of the information they needed. For example, participant 14 stated: “*I often check the Chinese websites if I need to know something*.” However, at the same time they questioned the accuracy of the information they retrieved from these websites. Participant 14 shared: “*Sometimes I wonder about the correctness [of the information of the website] because the information is inconsistent*.” Many of the participants felt they needed to confirm the information with their professional health care providers.

### The merits of the Canadian healthcare system

Although most women who were cared for by obstetricians complained about the lengthy transportation and long wait to see their physicians, they were satisfied with their care. Participants 7, a multipara, felt that the access to her obstetrician in Canada was better than in her country of birth. She stated: *“Back home I had to get up in the early morning in order to line up at the registry counter in the hospital to make an appointment with the doctor.”* This woman further explained that in order to see her doctor at 9 am she had to leave home around 5 am to draw a number from the numbering machine at 7 am. She then waited another hour to make an appointment at 8 am when the registry office opened.

Some women positively commented on the high level of privacy they were afforded by their maternity care providers in Toronto compared to their country of origin. Participant 13, who had previously given birth in her country of origin and now in Canada described *“Here in Canada it is better because it’s one-to-one when your doctor examines you at prenatal visits. Back home there were often some other women waiting inside the examining room and overheard the conversation between you and your doctor.”* She also preferred the cleanliness of the examining table, the sofa for the father of the baby in the labour and delivery unit, the single room maternity care and the caring attitude of the nurses in Canada compared to her experience in her country of origin.

### The need for culturally sensitive care

Several women complained about the cultural insensitivity of intrapartum and postpartum hospital maternity care. One participant complained that the nurses applied an “ice pack” to her swollen perineum. In Chinese tradition a woman should not touch anything cold after birth. The participant shared that she took the ice pack away as soon as the nurse left the room. Further cultural insensitivity was noted by participants who complained about the cold beverages they were given after birth. Ingesting cold foods after birth is based on the same traditional taboo as the ice pack. The participants however did share that they appreciated the hot soup and tea the nurses provided.

### The emergence of alternative supports and the use of private services

The majority of the participants had limited social support and few interpersonal networks in Canada because they and their husbands were without extended family in Canada. Eight participants invited their parents, parents-in-law or sister-in-law to visit to Canada to help them adjust to the physical and psychological challenges of being a new mother, and support them to “do the month”. Participants who had family members visit after the birth reported receiving instrumental support. Family members did household chores, cooked meals and bathed the baby for the participants. Some participants recalled the benefits of staying in the Zuo Yue Zi (postpartum) centres after birth in their country of birth where they were cared for and provided with postpartum teaching about self and infant care. This type of service is popular in Vancouver but not in Toronto. Five participants hired a “Yue-Sao” to take care of them and their baby at home. The responsibilities of the Yue-Sao included but were not limited to cooking specific foods for the mother, bathing the baby, taking care of the cord, cleaning the mother’s clothes and sometimes the house. Participant 11 stated: *“My own mother couldn’t come due to a visa issue and my husband didn’t know how to cook, so we hired a Yue-Sao. She stayed three hours every morning for a month to cook Zuo Yue Zi meals for me. She was a nurse back home, so she is professional and knowledgeable. She is my consultant for postnatal practices.”* However, two participants were alone after their infant’s birth with no husband or relatives to help them. They could not financially afford to hire someone to help them. Participant 8 came to Canada as a refugee and she stated: *“My parents could not come. I have to wash clothes and cook meals for my older son and myself.”*

Participants shared that their friends were very valuable supports for them, especially their pregnant friends. They shared information about selecting a good obstetrician, where to buy clothes, car seats and cribs. If their friends’ English was good enough, they would accompany the participants to the prenatal visits and labour and delivery unit to serve as a private interpreter. Participant 9 described the assistance she got from her friend: *“We [the participant and her mother] rushed into the hospital and my friend met us in the labour and delivery unit to translate what the nurses said to me. It was 3 o’clock in the morning. She was really kind to help me out.”* The participant was separated from her husband at that time and her friend went with her to every prenatal visit.

Husbands played an important support role for participants during the postpartum period, especially when they didn’t have extra helpers. However, many husbands were unsure of how to provide support because they were born under the “one-child” policy and were overprotected by their own parents. According to the participants their husbands needed to learn how to become protectors and supporters to their wives. Participants 6 appreciated what her husband did for her but said: *“It’s only two of us here (in Canada). My husband can only cook simple meals, such as chicken soup. So I started to cook two weeks after delivery. I am too inpatient to teach him to cook for me.”* Without any other helper she not only needed to take care of her baby but also had to do all the house chores.

## Discussion

Similar to other Canadian findings, Chinese women in this study preferred healthcare providers who shared their own background [15,17,27]. It is noteworthy that in our study women originally showed negative attitudes toward midwifery care. However, those women who were cared for by midwives were very positive about the care they received, which is consistent with the results found in a pan Canadian study [24]. The negative attitude of Chinese women toward midwifery care can be attributed to: (a) the view that childbirth is fraught with complications; (b) the lower quality of education and training for midwives compared to obstetricians; (c) the modernization of maternity care; and (d) the high caesarean section rates [20]. As a result, the general public in China and Taiwan mistrusts the practice of midwives [20,22].

Another noteworthy finding of this study was the emergence of alternative supports and the use of private services. Privately paid informal (no Canadian training or certification) postpartum support persons such as the Yue-Sao described by women in this study are a relatively new phenomenon in Canadian society [32]. The Netherlands have a similar postpartum support provider to the Chinese Yue-Sao called “Kramversorgers” who are funded by the state and have been an entrenched part of maternity care for many decades. Canada is starting to see more postpartum support providers like postpartum doulas available to those who can pay for the service. There definitely appears to be a need for high quality postpartum support, especially among immigrant women with limited or no family support. The development of Zuo Yue Zi centres in Toronto is in the initial stage; however, the service is expensive and the quality is questionable [32]. It is important that the government ensure that professional standards are in place to certify that the postpartum support providers emerging in Canada provide high quality safe pregnancy, childbirth and postpartum support.

Family members play an important role in a woman’s postpartum period; however, immigrant women often lack family support [6,27]. Similar to the findings of previous research several participants in this study shared the difficulties they encountered in successfully getting visitors’ visas for family members from abroad to come to help them during the challenging postpartum period [6,7]. Adequate social support is extremely important during challenging life transitions like childbirth and is linked to better health outcomes. Poor social support is linked to poorer health outcomes, higher rates of postpartum depression and greater difficulty adjusting to all the changes that come with childbirth [27]. Canadian policy makers should consider the development of policies to enable and expedite the successful application of visitors’ visas to family members from abroad who can provide needed support to immigrant women during the postpartum period. The Super Visa introduced by the Canadian Government in 2011 is a good solution for family reunification, which allows parents and grandparents of Canadian citizens and permanent residents to visit their family in Canada for up to two years without needing to renew their status.

Interestingly the Chinese women in this study learned how to navigate the Canadian health care system to identify maternity care providers they wanted to look after them. Although they could not self-refer to the physician of choice as they could in their country of origin [15], the women developed strategies, including advice from friends and the internet, to identify and request an obstetrician who met their cultural and language needs. This was likely possible because of the availability of Chinese speaking obstetricians within the Greater Toronto Area [19] and may be very different in other regions of Canada. The participants in this study were also willing to put up with the inconvenience of travel and long waiting times to see the doctor in order to have a Chinese-speaking obstetrician. This finding is similar to another Canadian study that looked at Chinese immigrant preferences regarding their family physician, i.e. Chinese immigrants preferred to go to Chinese-speaking family physicians even if English-speaking physicians were much closer in proximity to their homes [15].

Continuity of maternity care provider throughout pregnancy and childbirth was important to women in our study. Several participants shared that they were willing to pay an extra fee to have their obstetrician at their birth. The willingness to pay for continuity of service provider may reflect the socioeconomic status of the women who participated in this study. It may also reflect the differences in health care systems, where in their country of origin they expected to pay out of pocket to get the desired service from their obstetrician. This type of extra billing is not allowed under the Canada Health Act. A standardized private “medical” service could be enacted, so that those who prefer the same provider throughout the entire pregnancy can pay for this service.

Consistent with several Canadian studies, participants in this study commented that their obstetricians were too busy to provide detailed information regarding pregnancy, especially the Chinese obstetricians because they were too popular to spend more time with their patients [1,4]. Although participants received little information from their physicians, they were able to find other sources of information within their communities such as community health centres. Pregnancy-related pamphlets were available to the women in English and/or Chinese in many community health centres. Eleven participants attended prenatal classes and found the classes were very useful. It is noteworthy that internet played a major role in searching for information. Participants not only searched for pregnancy-related information on the internet but also shopped around for information about the reputation of a doctor or hospital. It is also noteworthy that although all of the participants had at least a college diploma or degree that were completed back home, they still preferred to receive information from Chinese sources. The use of Chinese resources may increase the barriers for these women to find local health services available. This barrier may be overcome if local health services information is provided on local Chinese language websites and other social media. Another issue that needs to be examined in the care of Chinese women is the quality of pregnancy, childbirth, and newborn care information women got from Chinese websites. The women themselves expressed concerns and uncertainty about the quality of the information.

Maternity services which meet the needs of pregnant women and new mothers are very important to optimize maternal and infant health [6-8,11]. Women’s satisfaction with maternity services is an important component of optimizing maternal health [10]. Overall the women in this study were satisfied with the maternity services they received. At the same time, the results of this study demonstrate that most of the women still adhered to some of the Chinese traditional practices related to childbirth, indicating that ethnic and cultural considerations are important when providing care to immigrant women. Care can often be adapted to be culturally appropriate. Nurses, for example, could easily adjust their care of Chinese immigrants to provide hot drinks rather than iced juice, and provide alternate comfort measures to ice packs to ease perineal pain and promote healing.

This study has several strengths. The qualitative approach allowed the researchers to hear the women’s stories. In addition, the fact that the women could be interviewed in their own native language allowed the women to tell their story without the hindrances caused by a language barrier. The study was further strengthen by the fact that TY is fluent in Mandarin, did the interviews, and translated and co-analyzed the data with colleagues. One of the limitations of this study is that participants were well educated. Thus the findings may not be transferable to the general Chinese population living in Canada. Further research using a more socioeconomically diverse sample of pregnant Chinese immigrant women is recommended.

This study has several implications. Immigrant Chinese women preferred same-language-speaking healthcare providers for their maternity care. Health services should be based on the needs of women. Producing more Chinese healthcare providers may be unrealistic and inefficient to address Chinese women’s concerns. However, health care providers who are open and sensitive to cultural diversity are needed within the health care system. This means that healthcare providers need to be aware of immigrant women’s needs and show a commitment to excellence in service provision; this includes offering interpreters and when possible providers who speak the women’s language. Women in this study expressed satisfaction with midwifery care although they were not initially in favour of this type of care. It is essential that efforts should be made to educate the public including immigrants about the high quality maternity care provided by Canadian midwifery services.

Continuity of maternity care providers is extremely important to many women. Strategies should be examined to improve the ability of maternity care providers to provide continuity of care throughout pregnancy and childbirth.

Our study findings indicate that some Chinese immigrant women are employing unregulated and potential untrained postpartum support providers. This may be partly due to the inability for some immigrants to access informal social support from family members abroad, and limited social networks in Canada. Research is needed to develop knowledge about the scope and quality of services received from these postpartum support providers. There likely also is a need for government subsidized postpartum support for immigrant and Canadian born new mothers who cannot afford to pay for private help. Efforts should be made to regulate postpartum support services and the new growing business of Zuo Yue Zi centres, to ensure consumers are receiving safe and good quality services.

## Conclusion

The results of this study provide new knowledge and understanding of immigrant Chinese women’s experiences in accessing maternity health services within a large metropolitan Canadian city. Participants described two unique experiences within the themes: preference for linguistically and culturally competent healthcare providers, with obstetricians over midwives, and the emergence of alternative supports and the use of private services. Few studies of immigrant maternity service access have mentioned these experiences which may be linked to cultural difference. Further investigation with women from different cultural backgrounds is needed to develop a comprehensive understanding of immigrant women’s health services needs during pregnancy, childbirth and the postpartum period.

## Competing interests

The authors declare that they have no competing interests.

## Authors’ contributions

TYL, conceived of and designed the study, analyzed the interview data, interpreted the result and drafted the manuscript. CKL, OW and NK participated in the design of the study, helped to analyze the data and interpret the results as well as critically reviewed the manuscript. YCL and CCL helped to analyze the data and interpret the results. All authors read and approved the final manuscript.

## Pre-publication history

The pre-publication history for this paper can be accessed here:

http://www.biomedcentral.com/1472-6963/14/114/prepub
